# Synthetic biology and therapeutic strategies for the degenerating brain: Synthetic biology approaches can transform classical cell and gene therapies, to provide new cures for neurodegenerative diseases

**DOI:** 10.1002/bies.201400094

**Published:** 2014-08-06

**Authors:** Carmen Agustín-Pavón, Mark Isalan

**Affiliations:** 1 Department of Life Sciences, Imperial College LondonLondon, UK

**Keywords:** artificial cell systems, genome editing, neurodegeneration, synthetic proteins

## Abstract

Synthetic biology is an emerging engineering discipline that attempts to design and rewire biological components, so as to achieve new functions in a robust and predictable manner. The new tools and strategies provided by synthetic biology have the potential to improve therapeutics for neurodegenerative diseases. In particular, synthetic biology will help design small molecules, proteins, gene networks, and vectors to target disease-related genes. Ultimately, new intelligent delivery systems will provide targeted and sustained therapeutic benefits. New treatments will arise from combining ‘protect and repair’ strategies: the use of drug treatments, the promotion of neurotrophic factor synthesis, and gene targeting. Going beyond RNAi and artificial transcription factors, site-specific genome modification is likely to play an increasing role, especially with newly available gene editing tools such as CRISPR/Cas9 systems. Taken together, these advances will help develop safe and long-term therapies for many brain diseases in human patients.

## Introduction

*‘*Our pleasures, joys, laughter, and jests arise from no other source than the brain; and so do our pains, grief, anxieties, and tears.’ Two millennia after Hippocrates first acknowledged this simple truth, understanding how the brain works is still one of our major challenges. Indeed, two ambitious initiatives are currently devising tools for imaging and controlling brain activity, ultimately to create a working computational model of the entire brain 1. Although considerable scientific efforts are being made towards making the 21st century the age of the brain, we still seem to have caught just a glimpse of the function of the most complex organ in the human body.

Understanding the healthy brain is one goal, working out how to treat it when it becomes diseased is quite another. Although human lifespan has increased as standards of living have risen, the incidence of brain diseases has increased as a consequence 2,3. Brain pathologies include neuropsychiatric conditions, such as depression, as well as neurodegenerative diseases – the former being a risk factor for the latter 4. Taking both types of disorders together, up to one third of the European population suffers from brain diseases every year 5.

**Figure 1:**
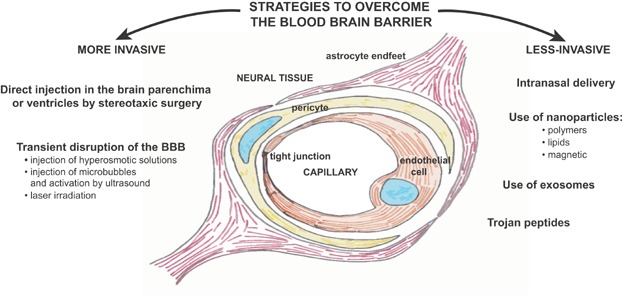
Overcoming the blood brain barrier for therapy. The sketch shows the cellular components of the blood brain barrier (BBB), which isolates and protects the neural tissue. The barrier prevents most drugs and therapeutic molecules from reaching their target sites in the central nervous system, when administered peripherally. Several strategies can be used to overcome the BBB, although these vary in their invasiveness. Recent developments include the use of nanoparticles and ‘Trojan peptides’ that naturally pass through lipid membranes to deliver their cargo. For a recent review of advances in delivery systems to the central nervous system, see 99.

Treating brain degeneration is now more necessary than ever, but it is especially difficult for a number of reasons. Putting aside our limited understanding of neural circuit functions, the intricate mixture of cell types populating the brain, the difficulty of targeted delivery, and the problem of designing drugs capable of crossing the blood brain barrier effectively (Fig. 1), we still face the daunting problem of rewiring and restoring the neural circuits when some of their components are lost as a result of degeneration. To circumvent this problem, it is critical to make the earliest possible interventions, ideally before neurodegeneration takes place. In other words, the focus should be in targeting pathophysiology rather than pathogenesis 6.

Discovering early biomarkers of disease is also essential, so as to improve diagnostic tools and to identify early symptoms. For example, in most common neurodegenerative conditions, early psychiatric symptoms such as depression and anxiety may precede the onset of neuronal loss by one or two decades. Some of these early symptoms are already being tested in the clinic for diagnostic potential 7,8. Metabolic and molecular biomarkers could also enable accurate predictions before the clinical onset of the disease, but they need extensive validation, and they raise ethical issues because of the unavoidable uncertainties associated with prediction 9.

Even if efficient early diagnosis were possible, we currently lack effective treatments for the degenerating brain. Most available drugs merely alleviate symptoms, without stopping disease progression or treating the underlying causes. It is therefore essential to devise novel, efficient therapeutic approaches to overcome these challenges; and, fortunately, some new potentially game-changing ideas are in the pipeline. These include the tools of synthetic biology, such as protein and gene network engineering 10,11, gene targeting 12,13 and genome editing 14–16.

In this review, we will look at the application of these tools to advance gene and cell therapy for neurodegenerative diseases. The discipline of synthetic biology is emerging with the promise of building standard, programmable and reusable parts for engineering biological systems 17. It is beyond our scope here to review the entire field, which is diverse. Very briefly, synthetic biology includes topics such as: engineering synthetic transcription, signalling and patterning networks 18,19; engineering metabolic networks to produce useful biomolecules (e.g. the malarial drug artemisinin 20); engineering multicellular systems (e.g. cellular logic gates and computers 21); building synthetic biomachines (e.g. swimming ‘jellyfish’ made from heart cells 22).

**Figure 2:**
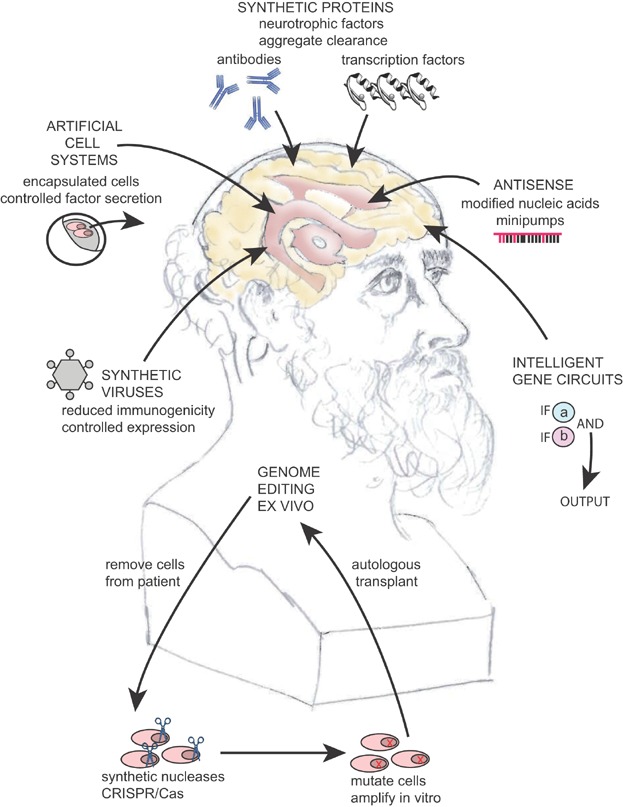
Overview of synthetic biology approaches for tackling neurodegenerative diseases. The sketch shows a marble bust of Hippocrates, who recorded some of the earliest insights about the functions of the brain. Note that many of the approaches can be used in combination.

Synthetic biology can contribute to gene and cell therapy for neurodegenerative diseases in several ways (Fig. 2). First, it can be used to modify classical gene therapy vectors for avoiding the immune system and yet for expressing therapeutic transgenes at the appropriate time, location, and quantity, to achieve long term therapeutic benefit. Second, synthetic biologists can design new molecules to target and modify expression in a tightly controlled manner. Pathogenic genes can be targeted, while taking into account their interactions in a given gene network. Genome engineering can be performed ex vivo in cells from different sources – including patient-specific cells and induced-pluripotent stem cells – or in vivo. Third, synthetic biomolecules can be designed to interact and clear aberrant proteins. Finally, it is possible to rewire genetic circuits in cells to build robust artificial systems that perform tasks of interest. These can then be delivered to the patient in the form of controllable microencapsulated cells. By combining some of these new approaches with classical gene therapy, we may obtain some urgently needed solutions for the problems of neurodegenerative diseases.

## How will gene therapy conquer neurodegenerative diseases?

The idea of gene therapy is beautifully simple: by repairing, replacing or modulating the activity of a diseased gene, you should be able to target the underlying causes of the pathology directly. Gene therapy approaches typically deliver genes or gene modulating factors (e.g. RNAi 23) with viral vectors. Standard vectors for use in the brain are adeno-associated viruses (AAVs) 24 and lentiviruses 25; these are modified to infect cell subtypes efficiently while addressing safety concerns by being non-replicating. Single gene diseases are easier to target and, in the context of neurological diseases, these include the metabolic disease of metachromatic leukodystrophy 26 and Huntington's disease (HD) 27. However, even single gene diseases are not always straightforward to tackle, because we often have a limited understanding of the multiple interactions of single genes embedded within larger disease-causing networks.

Neurodegenerative diseases such as Parkinson's (PD), Alzheimer's disease (AD), or amyotrophic lateral sclerosis (ALS), are generally complex (Box 1), hence making it difficult to choose a single gene to target, – although several trials are in development (Table1). The preferred approach so far has therefore been that of neuroprotection, via the administration of neurotrophic factors (factors that promote the growth, survival and maintenance of neurons) or by promoting their synthesis 6. Cell transplantation of bioengineered cells secreting neurotrophic factors is another promising strategy 28,29, but requires overcoming problems such as the limited distribution of factors in brain tissue, and the limited controllability of the release (Table1). Synthetic biology has the potential to improve these approaches both by modifying the gene delivery vectors and by providing new payloads to control gene expression.

**Box 1**

**Common and ‘rare’ neurodegenerative diseases**

Parkinson's and Alzheimer's disease are two of the most common neurodegenerative diseases of the brain. Both can be idiopathic or familial, and a number of different genes have been related to each condition. For AD, these include amyloid-β, presenilins, tau, and apolipoprotein E. For PD, genes include α-synuclein, parkin, or Leucine-rich repeat kinase 2. The multifactorial nature of these diseases makes them intrinsically more difficult to understand and to treat.

By contrast, HD has a single cause, but occurs more rarely with a frequency of approximately 1:10,000 people. HD belongs to a family of nine neurodegenerative disorders known as polyglutamine diseases, caused by the expansion of glutamine stretches coded within several unrelated genes. HD shares some features with PD and AD, including onset in late adulthood (for shorter polyglutamine expansions), selective neuronal vulnerability to disease-related proteins and abnormal protein processing and aggregation. Thus, HD is gaining importance as a model disease for developing therapeutic strategies that might translate across to the more common neurodegenerative diseases of diverse aetiology 27.

Similarly, whereas most of the cases of the motor neuron disease ALS are sporadic, the rare disease spinal muscular atrophy (SMA) is monogenic, making it an ideal candidate for gene therapy studies 100. SMA has a similar prevalence to HD, and can be caused by several mutations in the autosomic Survival Motor Neuron gene (*SMN1*). These include intragenic deletions, nonsense and point mutations, which reduce the production of SMN protein.

**Table 1:** Recent clinical trials illustrating strategies in gene and cell therapy for neurodegenerative diseases, showing trial outcomes and potential improvements from synthetic biology tools

Disease	Therapeutic approach	Outcome	Reference	Improvement with synthetic biology tools
AD	Antibody against amyloid β	Safety	110	Improved specificity, tailored antibody engineering
Encapsulated cells delivering NGF	Safety, low delivery levels after retrieval	29	Tightly controlled delivery levels
PD	Transplantation of levodopa-producing cells	No improvement	111	Improvement in delivery and distribution
Gene therapy: administration of neurotrophic factor	Safety	24	Improvement in delivery control and distribution
Gene therapy: administration of rate-limiting enzyme for dopamine	Safety and short term efficacy	112	Improvement in delivery and distribution
Transplant of fetal cells	Long term improvement in two patients	113	Synthetic non-fetal stem cells
SMA	Antisense oligonucleotides for RNA editing	Safety and dose-dependent muscle function improvement	45	Fully synthetic antisense oligonucleotides

As noted in the main text, the outcome of the treatments might critically depend on early administration, i.e. growth factors might be able to slow disease progression, but they will not rescue neuronal death. AD, Alzheimer's disease; PD, Parkinson's disease.

## Modifying gene therapy vectors reduces host clearance of therapeutic transgenes

Before discussing the possible genetic payloads that will be used in the next generation of gene therapy studies, it is important to consider the problem of long-term therapeutic expression of transgenes in the brain and vector design. Dramatic problems, such as the sudden death of a young patient in a gene therapy trial, as a direct consequence of viral vector infusion 30, have driven researchers to improve the safety of the viral vectors, by reducing the immunogenicity of vector capsid proteins 31. Another well-documented adverse effect has been insertional mutagenesis – the development of cancerous cells after insertion of the vector in the host genome 32. As a result, researchers have worked to reduce genomic insertion capabilities 33. Nevertheless, the development of increasingly safe vectors does not necessarily imply successful gene therapy, for it is also necessary to consider the toxicity and immunogenicity of administered genes. A striking example of this issue has been shown recently in a series of papers using rodents and non-human primates, simply using the common reporter gene GFP 34–36.

GFP is ubiquitous in modern biological research, and on the whole no toxicity is reported. However, it has been demonstrated that brain injections of an AAV vector expressing GFP leads to a strong immune response against the foreign protein, and neuroinflammation 3–4 weeks post-injection, causing significant neuronal loss 35,36. In addition, in rats, the infusion of a human gene leads to even stronger inflammatory responses, lasting at least 8 weeks after injection, with high neuronal loss 34. Thus, there is the risk that simply administering transgenes coding for foreign or synthetic proteins could trigger immune reactions in the brain.

To reduce this risk, it is possible to use bioinformatics tools for designing non-immunogenic constructs with fewer predicted epitopes 37. The resulting genes can be synthesised at a DNA level, including suitable host-optimised codons as well as coding host-like protein sequences. It should be noted that some foreign therapeutic transgenes will be easier to host-optimise than others. For example, out of the various DNA targeting systems (discussed in detail in *Genome editing with artificial nucleases*, below), only zinc fingers (ZFs) are native to mammalian systems. ZFs are thus easier to modify to remove immunogenic epitopes than, say, the prokaryotic TALE and CRISPR/Cas systems 38–40 (Table2).

**Table 2:** A comparison of genome editing tools

Structure and mechanism of DNA recognition	Origin	Used since	Pros	Cons
Zinc finger nucleases. Single aa within each ZF helix bind single DNA bases, each finger recognizes 3–4 bp 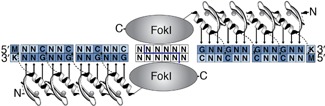	Eukaryotic transcription factor motifs	2001^16^,^114^,^115^	Low toxicity and immunogenicity in mammalian cells Economical size: bind ∼1 bp/10 aa Can concatenate to make long chains.	Difficult to engineer Less good at binding AT-rich DNA Need to design overlap between fingers
TALENs (Transcription activator-like effector nucleases). Two-aa residues within each ∼34 aa unit specifically recognise 1 bp 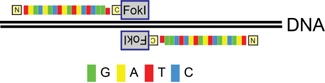	Prokaryotic host defense mechanism *Xantomonas* plant pathogen	2010^60^,^116^	Easy to engineer Full modularity to make long chains	Large and repetitive constructs: bind ∼1 bp/33 aa Less good at binding G-rich DNA Immunogenicity
CRISPR/Cas9 (clustered regulatory interspaced short palindromic repeats). RNA serves as a guide for the Cas9 nuclease, recognising ∼20 bp 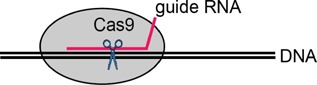	Prokaryotic ‘immune’ system against bacteriophages e.g. *Streptococcus pyogenes*	2013^105^,^117^	Quick and easy to bind new targets No protein engineering High efficiency	Lower specificity: ∼14/20 bp per complex Can use paired complexes for higher specificity, but not full concatenation Immunogenicity

aa, amino acid; FokI, nuclease domain from FokI restriction enzyme.

To ensure the safety and success of synthetic foreign transgenes in the brain we need to carry out long-term studies and model the possible off-target effects of the introduced transgenes. Even when the outcome is simply the repression of a mutated gene causing the disease, we need realistic models of how the perturbation in the diseased network affects the organism. Reversion of the system to its original ‘state’ is not a guaranteed outcome of expressing a therapeutic gene. In the case of neurotrophic factors, an additional level of modelling complexity is added by the fact that they are secreted, and will affect multiple cells, including non-transduced ones. Overall, synthetic genetic constructs need to be engineered sympathetically to the host organism, ideally with some systems-level understanding of the major gene expression and metabolic pathways prevalent in the target tissue.

## New synthetic payloads enable gene targeting, regulation, and editing

Moving beyond the general considerations of delivery vector design, synthetic biology tools can provide a series of new therapeutic payloads for treating neurodegenerative diseases. The general aim is to control the expression of target genes so as to prevent the loss of discreet populations of neurons, while enhancing the expression of neurotrophic factors to promote neuroprotection and self-recovery. Further to the RNAi-related developments of the last decade, synthetic biology has the potential to engineer transcription factors to control disease-related gene expression.

### ‘Killing the messenger’: RNA silencing and editing technologies are reaching maturity

One of the most tried and tested gene therapy strategies so far has been the silencing of mutated transcripts by means of antisense and RNA interference (RNAi) molecules. The use of chemically-modified nucleic acids to build stable small hairpin oligonucleotides (shRNAs) or microRNAs (miRNAs) has helped in this direction.

A pre-clinical study that successfully used this approach provided a safe, long-term treatment in several mouse models of HD. Targeting the mutant huntingtin mRNA gene with antisense 2′-*O*-methoxyethyl-phosphorothioate-modified chimeric oligonucleotides led to its degradation with RNAse H 41. This approach provided significant reductions of the mutant proteins for two months after treatment, in early-onset HD mouse models, and reversal of the motor abnormalities for six months after treatment, in a late-onset mouse model. Importantly, this treatment reduced the mRNA levels of huntingtin in a non-human primate for eight weeks after the termination of the treatment 41, highlighting its potential in translational medicine.

Similar modified oligonucleotides are already being translated to the clinic for spinal muscular atrophy (SMA) (Box 1). The Survival Motor Neuron gene (*SMN1*) is mutated in the disease, but the therapeutic strategy takes advantage of the presence of an almost identical gene variant (*SMN2*). *SMN2* contains an RNA splicing site that excludes exon 7 and produces a non-functional protein 42. However, antisense oligonucleotides can silence this splice site to promote the inclusion of exon 7, thus rescuing SMA. After successful pre-clinical studies in mouse models 43,44, several clinical trials are being developed 45 (Table1).

**Box 2**

**Genome editing for neurodegenerative disease models**

Synthetic nucleases have already been used to create brain disease models. For example, using ZF nucleases, PD, and AD model cell lines expressing mutations were created and, conversely, mutations in patient-derived induced pluripotent cells were corrected 101,102. Such techniques not only underpin the hope that we will be able to model diseases in a dish 103, building systems at will in order to increase our understanding, but they also pave the way for future therapies based on genome-edited patient stem cell transplants.
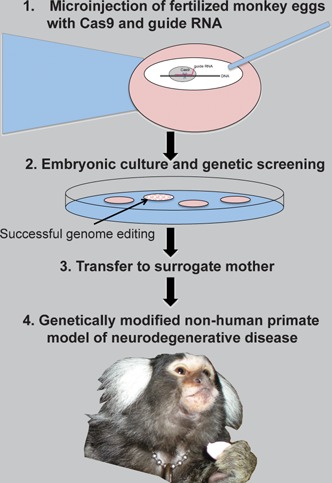

Although useful, cell culture approaches unfortunately cannot recapitulate the complex environment of an organism completely; factors such as immunity and cell-cell communication are of extreme importance. Moreover, the common inbred rodent models used are far removed from many aspects of human physiology in terms of both size and genetic diversity. Reflecting the move towards more personalised medicine, synthetic biology tools are helping to develop genetically modified animal models beyond the mouse, more rapidly and more efficiently 104–106. For example, using three plasmids coding TALENs, the *MECP2* gene was knocked down in monkey embryos with no detectable off-target mutations 107. *MECP2* deficiency is the cause of Rett syndrome, a severe neurodevelopmental disorder affecting only females (since the mutation is lethal for male embryos). A non-human primate model of this disease will help to shed light on the autism-related phenotype, and will enable more efficient and realistic pre-clinical trials. Potentially, this method could be used to create other neurodegenerative disease models in any non-human primate species, such as marmosets (see picture), an increasingly popular model species for neuroscience research due to its small size, ease of breeding and brain features comparable to humans 108,109.

As well as targeting mRNAs that code for disease-related proteins, recent studies are tackling non-coding RNAs that are also involved in pathogenesis 46. For example, a GGGGCC expansion is found in the promoter and first intron of the C9ORF72 gene, that makes a toxic non-coding transcript. This mutation is found in both ALS and frontotemporal dementia patients. Targeting the transcript with antisense oligonucleotides thus provides a promising common strategy for two different neurodegenerative diseases 47.

Although generally safe, some of these interference strategies are not devoid of peril for the organism. For example, shRNA has sometimes been found to be toxic in the brain 48–51, most likely because of saturation of the endogenous miRNA machinery, or because of off-target effects. A step forward in this strategy is the use of novel, fully synthetic, genetic polymers 52. These polymers, based on nucleic acid architectures not found in nature, can have the advantage of not being recognised by cellular enzymes and processing machinery, hence reducing the possibility of rejection, while retaining therapeutic potential.

## Synthetic transcription factors can flexibly regulate disease target genes

Whereas RNAi is generally limited to inhibiting target expression, artificial DNA binding proteins can be targeted to virtually any sequence, and can be fused to either transcription repression or activation domains 53,54.

For example, we designed and built a long poly-ZF protein able to bind to expanded stretches of CAG repeats, a mutation found in polyglutamine disorders, such as HD (Box 1). We fused our custom-made ZF to a KRAB repressor domain, and this synthetic transcription factor was efficient in repressing mutant huntingtin in human patient cell lines 12. The ZF was vectorised using AAV, and was delivered to mouse brains in the R6/2 HD model. This resulted in an acute reduction of the levels of mutant RNA and protein, and a concurrent delay in the onset of HD symptoms 12.

A similar approach was used in the neurons of the retina, as a treatment for retinitis pigmentosa. This disease causes blindness because of a mutation in rhodopsin. In a mouse model overexpressing mutated human rhodopsin, retinal injections of a viral vector carrying an engineered ZF, coupled to a transcriptional repressor, reduced transcription levels of mutated rhodopsin and prevented retinal degeneration 55.

Synthetic transcription factors have also been used to upregulate the transcription of endogenous neurotrophic factors. Generally, the virally-derived VP16 or VP64 transcription activation domains can be fused to any DNA binding domain to make activators 56,57. In a rat model of PD, a synthetic ZF coupled to the activator domain targeting the endogenous glial-derived neurotrophic factor (GDNF) promoter, was indeed neuroprotective 58. When following this strategy, it is important that the expression levels of the enhanced gene do not exceed the normal physiological range, since this risks toxicity 59. The therapeutic potential of artificial gene regulation is great, and, it is complemented by another level of synthetic biology: manipulating the genome at will.

## Genome editing with artificial nucleases: A geneticist's dream

Perhaps the most transformative technology to emerge in the last decade has been the possibility to harness the power of synthetic nucleases to allow genome-editing 16,60,61 (Table2). Nucleases make targeted double-stranded DNA breaks, which naturally induce knockouts (by the process of error-prone non-homologous end-joining) or knock-ins (by homologous recombination). Zinc finger nucleases (ZFNs), Transcription Activator like Effectors (TALENs) and, most recently, RNA-guided Clustered Regularly Interspaced Short Palindromic Repeat systems (CRISPR/Cas9), allow easy and efficient genetic engineering of mammalian cells. In particular, the latest generation of lentiviral Cas9 systems can reach genome modification efficiencies of >90% of targeted cells 14 – this would have seemed like science fiction just a few years ago.

Synthetic nucleases can be used to knock out, mutate, or repair a gene causing a given disease. One of the most promising aspects of this approach relies on the editing of human pluripotent stem cells for autologous transplants 62. An example is the recent success using ZF nucleases to delete the CCR5 HIV receptor in white blood cells, demonstrated to be a potential functional cure for HIV in patients 63.

Haematopoietic cell transplants, using engineered cells corrected for a mutation, have already shown promise for neurodegenerative diseases arising from metabolic defects, and therefore such diseases could also be treated with the latest generation of synthetic nucleases. Amongst these, lysosomal storage diseases are fatal conditions in which the accumulation of cell by-products in lysosomes leads to neurodegeneration. In metachromatic leukodystrophy, the deficiency of a single enzyme, arylsulfatase A, leads to fatality in infancy, within a few years of disease onset. Recently, a clinical trial has successfully used the autologous transplantation of engineered haematopoietic cells expressing the functional enzyme. These cells can migrate to the brain and clear the toxic accumulations of the enzyme substrate 26. The use of new genome editing technologies will greatly improve the efficiency and safety of genome editing with such aims, by integrating transgenes at specific loci, using homologous recombination.

The challenge of target specificity of synthetic nucleases is being actively researched and refined 64. Researchers are devising methods to improve specificity and avoid potential off-target effects, such as using DNA nickases rather than full cleavases 65. Nickases induce breaks in just one DNA strand, and they enhance homology-directed repair without activating non-homologous end joining repair. Although the CRISPR/Cas9 system is rapidly emerging as the easiest system to apply – simply requiring cloning of a guide RNA sequence to reprogram nuclease specificity – it originally had a weakness in terms of target specificity. Generally, >18 bp are required to specify a unique locus in the human genome. Although the CRISPR/Cas9 recognition complex covers 20 bp, ∼6 bp of the DNA recognition site tolerates mismatches, opening up the possibility of off-target effects. By combining two nickases, Ran et al. effectively doubled the specific binding target length, making unwanted off-target effects far less likely 66. The use of bioinformatic tools to predict off-target effects can also increase target specificity 67.

CRISPR systems are very practical, but some applications will require sustained expression, and in vivo studies have not yet investigated the long-term consequences of expressing these bacterial factors in the brain. The prokaryotic TALE effectors have also only been used for short studies: for example, when a light-controlled TALE was injected in mouse brain 68, animals were sacrificed only eight days post injection. By contrast, we have used eukaryotic ZFs to modulate the gene causing HD for up to four weeks in mouse brains 12, and can potentially make the entire protein sequence mouse-like, to avoid the immune system.

While in vivo techniques may be essential for complex diseases such as PD, in which discreet populations of neurons are lost, single-gene metabolic neurodegenerative diseases naturally suit the more tractable ex vivo approaches. As well as not having to consider immune rejection, one can deliver genome editing tools in the form of RNA ex vivo, hence eliminating the risks of long-term expression or unwanted random integration. Moreover, if stem cells can be modified, they can provide a long-term therapeutic benefit 62,63. Pluripotent stem cell transplant technology for the brain is still in its infancy, and it is still not clear how genome-edited neurons would be rewired correctly. While this major hurdle will require further research, genome editing already has other useful applications such as creating disease models (Box 2).

## Engineering approaches improve the clearance of aberrant proteins

One of the common hallmarks of neurodegenerative diseases is the aggregation of mutated proteins that possess seeding activity and can be transmitted in a prion-like fashion 69. A strategy to prevent aggregation is the administration of antibodies targeting these proteins. The advantage of this approach is that the antibodies can be delivered directly to the brain with minipumps, without the use of viral vectors, and without the need of modifying the genome, hence preventing undesired off-target effects. For example, a recent study in a mouse model of AD showed that selected antibodies administered chronically for three weeks to the brain ventricle decreased aggregation and improved cognitive deficits 70. These antibodies can also be administered as vaccines against prion-like diseases, and several clinical trials are on-going 71. Monoclonal antibodies are expensive to generate, however, and their production requires the use of living animals. Synthetic antibodies made by protein engineering offer a good alternative to naturally generated antibodies, with the possibility of improved specificity 72.

Antibodies are not the only peptides that can help clear mutant protein aggregates. Interactions with some natural proteins prevents aggregation and may be used as a therapeutic approach 73. Bauer et al. 74 tackled mutant Huntingtin protein in HD by designing a polyglutamine-binding peptide, fused to heat shock cognate protein 70 binding motif, such that it would promote degradation of aggregates by chaperone-mediated autophagy. Impressively, AAV delivery of this synthetic protein had a strong therapeutic effect in the R6/2 HD mouse model. Thus, synthetic biology principles can be used for the design and production of engineered proteins that will act with high affinity to target mutated proteins and prevent aggregation.

## Synthetic gene circuits: Creating intelligent genetic therapies

Synthetic biology can go beyond designed therapeutic proteins, expressed by simple promoters, and can achieve the precise control of gene expression by means of new promoter designs, gene switches, and circuit engineering.

Current gene therapy vector constructs utilise a relatively limited range of promoters to drive transgene expression. The combined CMV enhancer chicken β-actin (CAG) promoter and woodchuck hepatitis virus post-transcriptional regulatory element (WPRE) 75 are widely used for strong constitutive expression, whereas cell-type specific promoters are often used to limit transgene expression to subsets of cells 76,77. Since quantitative promoter characterisation is a key aspect of synthetic biology 78,79, new promoters should become available as a result.

Gene switches, based on small molecules or drugs administered to the patient, also have great potential for building conditional expression constructs in the brain. For example, the synthetic steroid hormone mifepristone – commonly used to treat Cushing's syndrome 80 – has been used as a gene switch in the brain. Mifepristone activated the production of GDNF, resulting in a neuroprotective treatment in a rat model of PD 81. Furthermore, in some cases, the switch molecule can also directly treat disease symptoms, resulting in double treatments 82.

An alternative and increasingly popular genetic switch is light-driven: optogenetics has revolutionized the field of neuroscience because of its precision and rapid kinetics 83. Light-controlled systems have successfully modulated neuron specific genes both in primary culture 84 and in the brains of mice: a TALE binding domain fused to plant cryptochrome 2 (sensitive to blue light) thus interacted conditionally with its partner protein CIB1, itself fused to an activator domain 68. Moreover, the use of fibre optics devices for gene control in the brain is feasible. Implanted electrodes for stimulation are already in use for PD patients 85, and experimental models in which neuronal activity is controlled in vivo by optogenetic techniques is achieving great refinement 86.

Moving from switches to full genetic circuits, perhaps the most exciting developments will come from intelligent constructs that integrate multiple inputs to get conditional outputs. Mammalian gene circuit engineering is still in its infancy, and most examples still focus on systems to make desired patterns of gene expression 19,87. However, several research projects are developing genetic logic gates, analogous to the gates used in electronics 10,11. For example, AND gates require two simultaneous activation inputs, such as the binding of heterodimer parts of transcription factors, to achieve conditional expression of a desired output gene (Fig. 1). Based on similar principles, cancer-detection circuits are being developed 13,88, and it is only a matter of time before these are adapted to gene therapy applications.

## Will artificial cell transplants achieve long-term neuroprotection?

One of the most ambitious aims of synthetic biologists is to create synthetic cells equipped with tightly regulated gene networks to transplant into host organisms. These would release therapeutic compounds in a controlled manner to help organisms to self-repair. Such cells need to be carefully isolated from the host to prevent immune rejection and undesired proliferation. This is achieved by encapsulation with a biocompatible, semipermeable material, such as alginate-poly-(l-lysine)-alginate, which allows the exchange of essential biomolecules and ions, while isolating the cells from the immune system of the host 82,89,90.

In recent work following this strategy, a biosensor cell was built that could sense a change in the neurotransmitter dopamine, which is involved in processes such as movement and motivation 91. The D1 dopamine receptor was coupled to an intracellular signalling cascade to activate the production of atrial natriuretic peptide (ANP), a natural anti-hypertensive molecule. Implanting these genetically engineered encapsulated cells in the peritoneum of hypertensive mice demonstrated that the production of ANP was enhanced in a controllable way by dopamine agonists or sexual arousal. This reduced and controlled the blood pressure of the mice in both situations 89.

Pharmacological and gene therapies can be combined for diseases with multiple symptoms. To treat metabolic syndrome, a chimeric TAAR (trace amine-associated receptor, coupled to G protein) was rewired to activate two genes controlling food intake, namely glucagon-like peptide 1 and leptin. The chimeric receptor responds to guanabenz, a drug clinically prescribed to treat hypertension. Microencapsulated cells bearing this system were implanted in the peritoneal cavity of mice. This double treatment was successful in attenuating several symptoms of metabolic syndrome in obese mice 82.

Encapsulated cells have long been used to release neurotrophic factors in the treatment of neurodegenerative diseases 90, but the main outcomes of those clinical trials have been the general safety of the implants, rather than strong therapeutic results. Problems have included the limited distribution of secreted neurotrophic factors, and fluctuating production and responses, caused by circadian rhythms. Mammalian synthetic biology researchers are building artificial oscillators, inspired by circadian clocks 92, and tunable secretion and cell-cell communication systems 19. In the future, we may build more complex sender-receiver systems, including feedback elements that enhance the distribution of secreted therapeutic factors.

As noted in the introduction, successful treatments for neurodegenerative diseases require molecular biomarkers for accurate early prediction. It was recently discovered that the depletion of ten lipids in peripheral blood predicts the occurrence of amnestic mild cognitive impairment or AD within 2–3 years, with over 90% accuracy 93. Similarly, an auto-antibody against a potassium channel, whose dysfunction is related to neurodegeneration 94, was found in multiple sclerosis patients before symptom onset 95. If such biomarkers could be linked to biosensor circuits in encapsulated cells 96, for the conditional production of neurotrophic factors, one could envisage predictive and protective strategies for neurodegenerative diseases. These would aim to delay onset and to slow disease progression, and would be targeted to susceptible populations.

Artificial cells can also be used to release specific factors for the promotion of self-repair. Adult neurogenesis is active in some specific niches in the human brain, and this process is dysregulated in neurodegenerative disorders 97. The recent discovery of adult neurogenesis in the human striatum 98 opens the exciting possibility of investigating the regulatory factors required for cell migration and differentiation. A deeper knowledge of the factors, and their roles in diseases like HD and PD, would allow the design of regulators to enhance these processes, perhaps in combination with neuroprotection. Another issue is that projection neurons (large neurons wiring main circuits) are lost in such diseases, while adult-produced cells only differentiate into interneurons (small neurons that modulate the main circuits) and glia. The challenges are to design regulators that could further influence the fate of the progenitors to differentiate into projections neurons, and to promote rewiring.

## Conclusions and outlook

Synthetic biology can provide many useful approaches for the rational design of therapeutic factors and intelligent systems to treat a variety of diseases, including targeting the most complicated organ in our body, the brain. We can already find examples of the design of factors for genome editing, the regulation of gene expression, protein targeting, and for biosensor cells able to release therapeutic molecules in a tightly regulated manner, both in response to endogenous signals or to drugs. With all these new tools, we will be able to repair or to complement the pathogenic genes in specific conditions, such as those with single-gene metabolic causes. For many diseases, we will need to establish combined protective and restorative actions. Harnessing natural processes to promote self-repair and an improved basic knowledge of the underlying development and circuitry of the brain will be essential for developing new synthetic therapies that act in concert with host physiology. Early detection and intervention is crucial for beating the devastation caused by neurodegeneration. Perhaps the most exciting long-term possibility is to link biomarkers to biosensors, to express effectors conditionally, and to reverse pathogenesis as early as possible. Although the work has begun, we still need a lot more research to design a safe and long-term gene or cell therapy based on synthetic biology principles. Using nature as our inspiration, we will continue to improve our intelligent synthetic designs.

## Abbreviations

AAV: adeno-associated virus
AD: Alzheimer's disease
ALS: amyotrophic lateral sclerosis
GDNF: glial-derived neurotrophic factor
HD: Huntington's disease
PD: Parkinson's disease
ZF: zinc finger
